# Dentate Gyrus Morphogenesis is Regulated by an Autism Risk Gene *Trio* Function in Granule Cells

**DOI:** 10.1007/s12264-024-01241-y

**Published:** 2024-06-22

**Authors:** Mengwen Sun, Weizhen Xue, Hu Meng, Xiaoxuan Sun, Tianlan Lu, Weihua Yue, Lifang Wang, Dai Zhang, Jun Li

**Affiliations:** 1 Peking University Sixth Hospital, Peking University Institute of Mental Health, NHC Key Laboratory of Mental Health (Peking University), National Clinical Research Center for Mental Disorders (Peking University Sixth Hospital), Key laboratory of Mental Health, Chinese Academy of Medical Sciences, Beijing, 100083 China; 2 Peking-Tsinghua Center for Life Sciences, Peking University, Beijing, 100871 China; 3 BGI-Beijing, Beijing, 102601 China; 4 PKU-IDG/McGovern Institute for Brain Research, Peking University, Beijing, 100871 China; 5 Institute for Brain Research and Rehabilitation (IBRR), Guangdong Key Laboratory of Mental Health and Cognitive Science, South China Normal University, Guangzhou, 510631 China; 6 Changping Laboratory, Beijing, 102299 China

**Keywords:** *Trio*, Autism spectrum disorders, Dentate gyrus morphogenesis, Neuron migration, Spatial transcriptomic sequencing

## Abstract

Autism Spectrum Disorders (ASDs) are reported as a group of neurodevelopmental disorders. The structural changes of brain regions including the hippocampus were widely reported in autistic patients and mouse models with dysfunction of ASD risk genes, but the underlying mechanisms are not fully understood. Here, we report that deletion of *Trio*, a high-susceptibility gene of ASDs, causes a postnatal dentate gyrus (DG) hypoplasia with a zigzagged suprapyramidal blade, and the *Trio*-deficient mice display autism-like behaviors. The impaired morphogenesis of DG is mainly caused by disturbing the postnatal distribution of postmitotic granule cells (GCs), which further results in a migration deficit of neural progenitors. Furthermore, we reveal that *Trio* plays different roles in various excitatory neural cells by spatial transcriptomic sequencing, especially the role of regulating the migration of postmitotic GCs. In summary, our findings provide evidence of cellular mechanisms that Trio is involved in postnatal DG morphogenesis.

**Supplementary Information:**

The online version contains supplementary material available at 10.1007/s12264-024-01241-y.

## Introduction

Autism Spectrum Disorders (ASDs) are a group of neurodevelopmental disorders with high heritability, defined as abnormal social interaction, communication, and restricted repetitive behaviors or interests [1–4]. The volume and/or structural changes of the hippocampus were reported in many cases with ASDs [5–9], especially the focal architecture distortion of the granule cell (GC) layer and ectopic distribution of GCs in the dentate gyrus (DG) [10, 11]. This evidence indicated the possibility that the maldevelopment of the hippocampus, especially the DG, might be involved in the pathogenesis of ASDs.

The developmental strategy of DG is unique and regulated by multiple molecules [12–14], including the tangential migration of neural progenitors from the neuroepithelium towards the hippocampal fissure to form a hub of proliferating cells called DG anlage during the perinatal period, and the radial migration of these progenitors from subpial zone (SPZ) to subgranular zone (SGZ) at early postnatal stage [15, 16]. However, the genetic risks of ASDs in DG dysplasia and the underlying neurobiological mechanisms were largely unknown [17].

Trio is one of the key guanine nucleotide exchange factors (GEFs), involved in regulating neuron migration and axon guidance [18–23]. It is also identified as a high-risk gene for ASDs with a large cluster of mutations [24–27]. Although previous studies have reported that Trio insufficiency in mice was related to the disorganization of GCs in DG [28], the underlying mechanism and the relationship between Trio-deficiency-induced DG malformation and ASD-like behaviors were not clarified.

In this study, we demonstrated that genetic deletion of Trio impaired the morphogenesis of DG by disturbing the postnatal distribution of postmitotic GCs, which further resulted in a migration deficit of neural progenitors. Furthermore, we revealed that Trio played different roles in various excitatory neural cells involved in postnatal DG development by spatial transcriptomic sequencing. The maldevelopment of DG induced by *Trio* deletion might be related to autistic behaviors including impaired social novelty recognition and increased stereotypic behaviors. Our finding revealed that *Trio* regulates the morphogenesis of DG and is involved in the pathology of ASD-related phenotypes.

## Materials and Methods

### Animals

The protocols for the use and care of all mice were approved by and performed according to the guidelines of the Animal Care and Use Committee of Peking University (Beijing, China). The mice were housed on a 12-h light-dark cycle and provided food and water. *Trio*^*fl/fl*^ mice were purchased from the Model Animal Research Center (Nanjing, China) as previously described [19]. Trio conditional knockout (cKO) mice were generated by crossing the *Trio*^*fl/fl*^ mice with mice expressing the Cre recombinase transgene under the control of the Emx1 and Nex promoter. Emx1-Cre and Nex-cre mice were generous gifts from C. Zhao (Southeast University, Nanjing, China) and Z. Qiu (Songjiang Research Institute, Shanghai Jiao Tong University School of Medicine, Shanghai, China), respectively. The mouse strains used in the present study are on C57BL/6 background.

### Experimental Design

For morphological tests, female and male mice were both used. Thirty-µm coronal sections from the rostral and caudal hippocampus were examined in at least three matched sections in each brain by an experimenter who is blind to the animal genotype. Three to five subjects were included in each group. During counting and scoring, the whole DG area was included, since cells in mutant DG were ectopically distributed. For the behavioral test, only male mice were used, thirteen to eighteen subjects were included in each group.

### EdU Injection

5-ethynyl-2′-deoxyuridine (EdU, Thermo Fisher, C10340) was dissolved in Saline with a concentration of 2.5 mg/mL. Mice were intraperitoneally injected or orally administered (mice that were younger than P7) at the dose of 50 mg/kg for pulse labeling or birthdating analysis.

### Tissue Preparation

Mice were perfused intracardially with saline, followed by 4% paraformaldehyde (PFA) in PBS after deep anesthesia with sodium pentobarbital (40 mg/kg, dissolved in saline). Brains were immersion-fixed in 4% PFA for 6−8 h at 4 °C and cryoprotected in 30% sucrose for over 48 h. For immunostaining, 30 µm coronal hippocampal sections were performed with a cryostat (Leica CM1950).

### Immunofluorescence

Brain sections were washed in PBS three times, permeabilized, and blocked with PBS containing 5%(wt/vol) BSA and 0.3% Triton X-100 for 30 min at room temperature, and incubated with primary antibody at 4 °C overnight. Sections were subsequently washed in PBS, incubated with a secondary antibody for 1h at room temperature, and washed with PBS. The following antibodies and reagents were used for immunostaining: anti-BLBP (Abcam, AB32423, 1:1000), anti-PAX6 (Covance, 901301, 1:200), anti-Tbr2 (Abcam, AB183991, 1:200), anti-CTIP2 (Abcam, AB18465, 1:500), anti-GFAP (Millipore, NE1015, 1:500), anti-prox1 (Millipore, MAB5654, 1:100), anti-NeuN (Millipore, MAB377B, 1:100), anti-Ki67 (Abcam, AB15580, 1:500), anti-caspase3 (Cell Signaling Technology, P42574, 1:500), Alexa Fluor 555 goat anti-rat IgG (Invitrogen, A31570, 1:1000); Alexa Fluor 555 goat anti-mouse IgG (Invitrogen, A21050, 1:1000); Alexa Fluor 488 goat anti-mouse IgG (Invitrogen, A11008, 1:1000), Alexa Fluor 555 donkey anti-rabbit IgG (Invitrogen, A31572, 1:1000); and Alexa Fluor 488 goat anti-rabbit IgG (Invitrogen, A11006, 1:1000), EdU imaging Kit (Thermo Fisher, C10340), Hoechst (Invitrogen 33342). Sections were imaged with a confocal microscope (Olympus FV1200, Japan), and analyzed by ImageJ software.

### RNAscope

The staining was performed by using the RNAscope Multiplex Fluorescent Kit (ACD 323100) with the probe of Trio according to the manufacturer’s protocols. Briefly, brain sections were fixed with PFA at 4 °C for 15min, and treated with antigen retrieval buffer at 95–100 °C for 10min before being incubated with protease for 30min at 40°C. After pretreatment, the sections were incubated with a hybridization probe for 2h at 40°C and then stained with fluorescent dye and DAPI. When co-staining with proteins, brain sections would be incubated with primary antibody at 4 °C overnight before hybridizing with a probe. Sections were imaged with a confocal microscope (Olympus FV1200, Japan), and analyzed by ImageJ software.

### Western Blotting

The DGs were dissected out and homogenized on ice in protein lysis buffer containing the following reagents: RIPA (50 mmol/L Tris [pH 7.4]), 1%TritonX-100, and protease inhibitors (Roche, 11697498001). Lysates were centrifuged at 12,000× g for 20 min at 4 $^\circ{\rm C}$. Supernatants were collected and boiled with 4× Laemmli sample buffer (Sigma-Aldrich) and loaded onto SDS-PAGE gels. After membrane transfer, rabbit anti-Trio (Santa Cruz, sc-28564, 1:500), rabbit anti-Gapdh (Cell Signaling Technology, D16H11, 1:3000) and HRP-conjugated secondary antibody (1:2000) were used for immunoblotting. Membranes were developed and imaged using an ECL chemiluminescence reagent (Thermo Fisher Scientific) and a Tanon 5200 Automatic chemiluminescence imaging analysis system (Tanon, Shanghai, China).

### Spatial Transcriptome

For tissue preparation, postnatal day 0 (P0) mouse brain tissues of male littermates were collected and embedded in prechilled Tissue-Tek OCT (Sakura 4583), and snap-frozen in liquid nitrogen. Cryosections were cut at a thickness of 10 μm in a Leica CM1950 cryostat. For stereo-sequencing, tissue sections were adhered to the Stereo-seq chip surface (10 mm × 10 mm capture areas, with DNA nanoball (DNB) containing random barcoded sequences) and incubated at 37 °C for 3−5 min. Where incubated, the same sections were stained with nucleic acid dye (Thermo Fisher, Q10212) prior to *in situ* capture and sequencing as described before [29]. For DG cell segmentation and annotation, we recognized and segmented the Dentate Gyrus region from the image of the nuclei acid staining section, and projected the staining image to the Stereo-seq chips of the same section. After alignment, we applied the BGI sitemap (https://www.stomics.tech/) to perform cell segmentation analysis. We characterized cell clusters by their expression levels of neuronal-specific genes (BLBP, GFAP, Tbr2, Prox1, CTIP2, and NeuN) with bin size of 30. For analyzing different gene and signaling pathways, we conducted differential gene expression analysis by Seurat (V4.1.1) in each cell cluster between wild-type and conditional knock-out mice. Genes with a fold of no less than 2 and an adjusted *P* value of less than 0.05 were retained. Volcano maps were constructed by the R package ggplot2 (version 3.2.0). To perform enrichment analysis for signaling pathways and gene ontology categories, we used the R package clusterProfiler (V4.4.4) and Gene Ontology (GO) database. Hypergeometric tests were performed and *P* values were adjusted using the Benjamin-Hochberg method, setting the *P* value and the *q*-value cutoff at 0.05. GO maps were constructed by the R package ggplot2 (version 3.2.0). In addition, ranked gene set enrichment analysis (GSEA) was undertaken by R package irGSEA (V1.1.3), to identify the cell clusters that were involved in migrating progress.

### Behavioral Tests

Animals were housed at a constant temperature of 24−25 °C on a 12 h/12 h light-dark cycle (lights on at 8:00 am), with food and water available *ad libitum*. Behavioral tests were performed on the light cycles with 5−10-week-old male mice as previously described [30]. The movement of mice in the three-chamber social interaction test, elevated plus-maze test, hole-board test, marble burying test, and tube test were tracked and recorded with the Etho Vision 8.0 video-tracking system (Nodules, the Netherlands). All animal procedures were approved by the Animal Ethical Committee of Peking University Health Center.

#### Three-Chamber Social Interaction Test

The chamber was a white rectangular apparatus (L× W × H = 60 cm × 40 cm × 22 cm), which was evenly divided into three parts by two transparent acrylic walls with an opened door (5 cm × 8 cm) on each of them. Two grid enclosures (7 cm in diameter and 15 cm in height) were placed in the ipsilateral corner of both the left and right chambers. A 6-week-old male mouse was allowed to explore the chamber freely for 5 min. After that, an age- and gender-matched stranger mouse (stranger1, S1) was placed in one of the enclosers, and the subject mouse was allowed to explore the chamber and socialize with S1 for 10 min. Then another novel mouse (stranger 2, S2) was placed into the ipsilateral enclosure, and the subject mouse would explore the chamber and socialize with mice in two enclosures for another 10 min. The time that the subject mouse spent in each part of the chamber and close social interaction (with the nose point within 2 cm of the enclosure) were recorded and calculated.

#### Hole-Board Test

A 15 cm elevated grayboard (40 cm × 40 cm) with 16 uniformly distributed holes (3 cm in diameter) was used for the hole-board test. During the test, a 6-week-old male mouse was placed on the center of the board, and the number of head-dipping behaviors in 10 min was analyzed. An “exploratory dip” was defined as any head dip into a hole different from the previous one. A “stereotyped dip” was a dip into the same hole as the previous dip. The increase in stereotyped dip numbers would be considered a sign of stereotyped behavior.

#### Marble Burying Test

A standard polycarbonate cage (L× W × H = 40 cm × 25 cm × 20 cm) was filled with unscented fresh bedding material to a depth of 6–7 cm and the level of bedding surface was flattened by inserting another cage of the same size onto the surface of the bedding. A total of 20 standard glass toy marbles (assorted styles and colors, 15 mm in diameter, 5.5 g in weight) were completely cleaned and dried before use and carefully placed on the surface of the bedding in five rows and four columns. At the beginning of the test, one male mouse was put into one of the corners of the cage, as far from the marbles as possible, and allowed to explore the cage undisturbed for 30 min. After that, the number of buried marbles was counted. A marble was considered buried if two-thirds of its surface area was covered by bedding. Each test session was conducted in a clean mouse cage with fresh bedding.

#### Light–Dark Box

The test was performed in the open field box (a clear box that L× W × H = 27.5 cm × 27.5 cm × 20 cm, MED Associates) with a dark box insert apparatus (L× W × H = 27.5 cm × 14 cm × 20 cm) so that the field was divided into a dark chamber and a light chamber on each side. While starting the test, a 6-week-old male mouse was placed at the boundary and its head towards the dark chamber. The subject mouse was allowed to explore the apparatus for 5 min freely, and the time that the mouse spent in the light chamber was used as an indicator of anxiety-like behavior.

#### Elevated Plus-Maze

The test was performed by using an elevated plus-maze with a central 5 cm × 5 cm platform with two 30 cm × 5 cm plus-shaped arms, and the enclosed arm was equipped with 15 cm-high walls. The maze was elevated to a height of 60 cm above the floor. The mice were placed on the central platform facing an open arm of the plus-maze. The time each mouse spent in the open arms during the total 5 min period was calculated and used for assessment of the anxiety-like behavior.

#### Open Field Assay

A plexiglass box (L× W × H = 27.5 cm × 27.5 cm × 20 cm, MED Associates) was used to perform the test. At the beginning of the test, mice were placed in the center of the box. The time that mice spent in the central zone of the open field in 10 min was recorded to evaluate the anxiety-like behavior of mice.

### Statistical Analysis

During testing and scoring, the experimenters were blinded to the genotype of the mice. No samples or animals were excluded unless specified in the experimental procedure. All data in accordance with the normal distribution were represented as mean ± SEM. The homogeneity test of variance was performed before the statistical test. Statistical Package for the Social Sciences 20.0 (SPSS, Chicago, IL, USA) and GraphPad Prism 7.04 were used for statistical analysis, *P* < 0.05 was considered as statistical significance.

## Results

### *Trio* Ablation in Progenitors Leads to a Smaller and Twisted DG

**Fig. 1:**
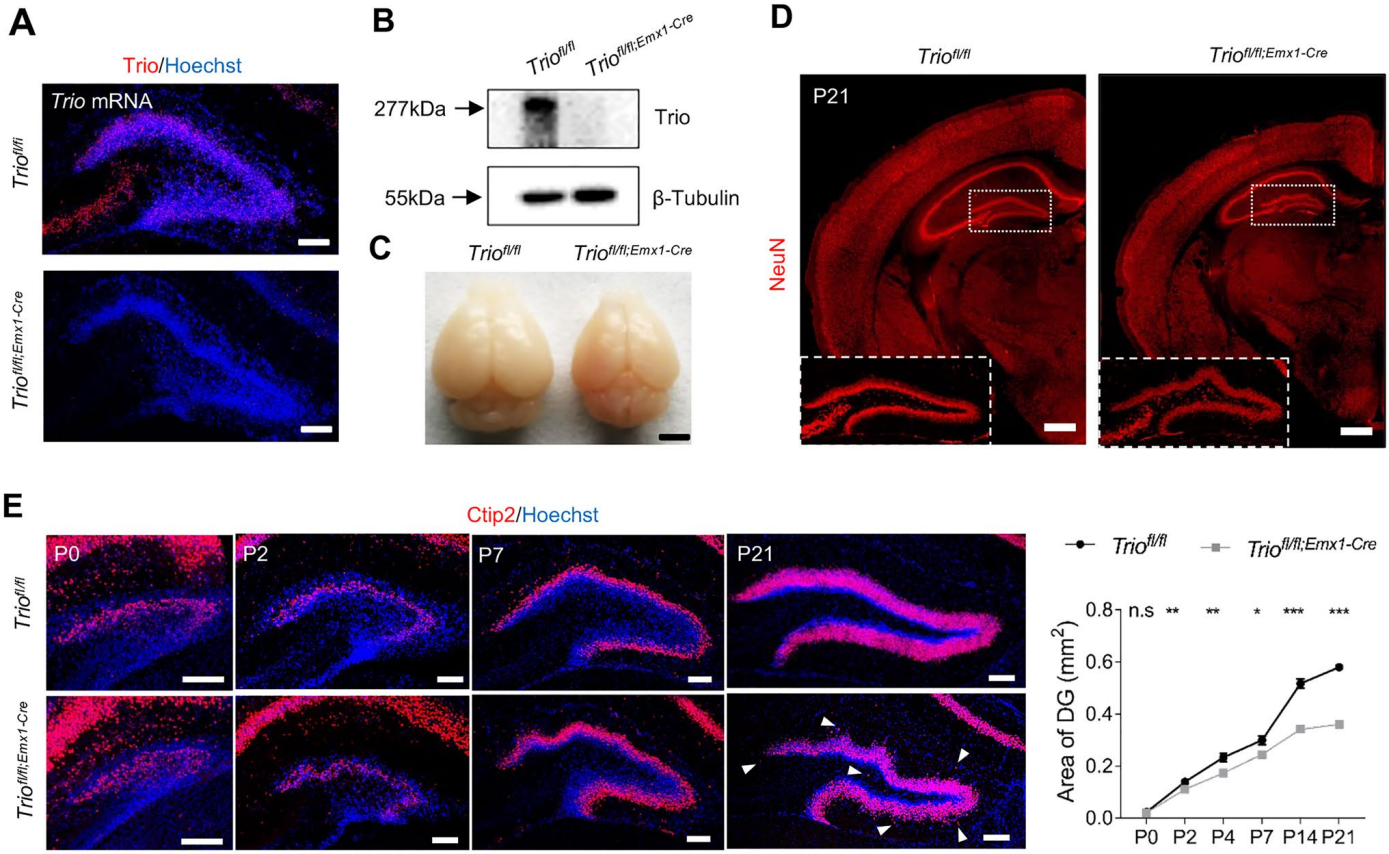
Deletion of *Trio* in forebrain neural progenitors leads to DG hypoplasia at postnatal stages. **A** *Trio* mRNA expression in DG from *Trio*^*fl/fl*^ mice and *Trio*^*fl/fl;Emx1-Cre*^ mice at P4. Scale bars, 100 µm. **B** *Trio* protein expression in HIP from *Trio*^*fl/fl*^ mice and *Trio*^*fl/fl;Emx1-Cre*^ mice at P21. **C** Brain size of *Trio*^*fl/fl;Emx1-Cre*^ mice was smaller at P21. Scale bars, 2 mm. **D** P21 coronal sections revealed abnormal morphology of DG in *Trio*^*fl/fl;Emx1-Cre*^ mice. Scale bars, 500 µm. **E** Morphological changes in Trio-deleted DGs at postnatal developing stages (left) and the area of the DG decreased in different levels in *Trio*^*fl/fl;Emx1-Cre*^ DGs (right) (*n* = 5 WT; *n* = 5 cKO). Arrowheads indicated the ectopic granule cells in *Trio*^*fl/fl;Emx1-Cre*^ DG at P21. Data were shown as means ± SEM.*P <0.05, **P <0.01, ***P <0.001; n.s., no significance, two-tailed Student’s *t*-test. Scale bars, 100 µm.

To determine the function of Trio in hippocampal development, we first detected the protein level of Trio in the hippocampus at postnatal developmental stages (Fig. S1A) and generated *Trio*^*fl/fl;Emx1-cre*^ mice to specifically disrupt Trio in progenitors in the forebrain (Fig. 1A, B). We found that the brain size of conditional knockout mice was smaller than that of control mice (Fig. 1C). Interestingly, the brain sections showed smaller hippocampus and DG, especially severely twisted suprapyramidal/infrapyramidal blades of DG (Fig. 1D) and zigzagged arrangement of GCs in cKO mice at P2, P7, and P21 (Fig. 1E), with several piles of GCs detected out of the GC layer (arrowhead). However, neither the arrangement of GCs nor the size of DG was affected at P0 (Fig. S1B), and the pattern of DG anlage was not significantly changed in embryonic and perinatal DG of *Trio*^*fl/fl;Emx1-Cre*^ mice (Fig. S1C, D). Thus, our data indicated that ablation of *Trio* in progenitors disrupted the morphogenesis of the DG GC layer at the postnatal stage.

### *Trio* Deletion Leads to a Dynamic Reduction and Disturbed Distribution of Progenitors and GCs in Postnatal DG

**Fig. 2:**
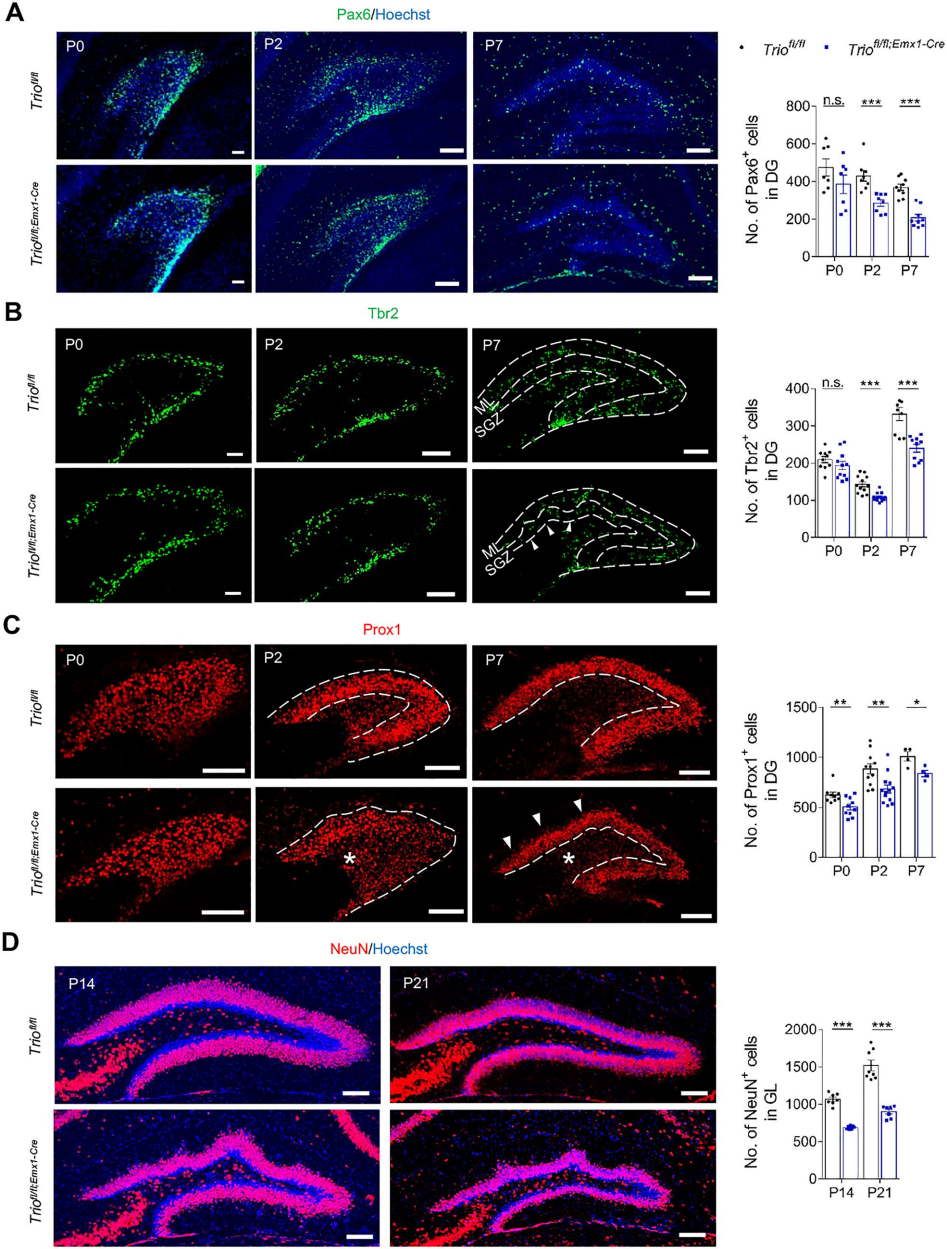
Dynamic reductions and disturbed distribution of progenitors and GCs in postnatal DG following *Trio* deletion. **A** The absolute number of progenitors in DG at P0 (*n* = 3 WT; *n* = 3 cKO), P2 (*n* = 3; 3), and P7 (*n* = 3; 3). Scale bars, 50 µm. **B** The absolute number of IPCs in DG at P0 (*n* = 3; 4), P2 (*n* = 4; 4) and P7 (*n* = 3; 3). The arrowhead indicated the missing IPCs in SGZ of *Trio*^*fl/fl;Emx1-Cre*^ mice. Scale bars, 50 µm. **C** The absolute number of postmitotic neurons in DG at P0 (*n* = 3; 4), P2 (*n* = 4; 4), and P7 (*n* = 3; 3). Dashed lines illustrated the potential borderlines of densely packed cell bands at P2 and P7. Stars indicated the ectopically distributed cells in the hilus region of *Trio*^*fl/fl;Emx1-Cre*^ mice at P2 and P7. Arrowheads indicated the loosely packed cells in ML of *Trio*^*fl/fl;Emx1-Cre*^ mice at P7. Scale bars, 100 µm. **D** The absolute number of GCs in DG at P14 (*n* = 3; 3) and P21 (*n* = 3; 3). Scale bars, 100 µm. Data were shown as means ± SEM. *P< 0.05, **P <0.01, ***P <0.001; n.s., no significance, two-tailed Student’s *t-*test.

To further clarify the cellular mechanism of DG malformation induced by *Trio* deletion, we labeled and quantified different types of cells at postnatal stages. We found that the total number of Pax6^+^ dentate progenitors (Fig. 2A) and Tbr2^+^ intermediate progenitors (IPCs; Fig. 2B) were significantly reduced at P2 and P7 but not P0 in *Trio*^*fl/fl;Emx1-Cre*^ mice. To further characterize the number of postmitotic GCs that were generated from progenitors, we performed Prox1 and NeuN immunostaining for immature and mature neurons, respectively. We found that the numbers of both Prox1^+^ and NeuN^+^ neurons were decreased in *Trio*^*fl/fl;Emx1-Cre*^ DG (Fig. 2C, D). In addition, the distribution patterns of progenitors and GCs were also disturbed in *Trio* deficiency DG. Progenitors settled in SGZ declined while many of them stayed in the molecular layer (ML) at P7 in *Trio*^*fl/fl;Emx1-Cre*^ mice (Fig. 2B, arrowhead), and GCs were loosely arranged with more GCs were scattered out of the granule cell layer (GL) in *Trio*^*fl/fl;Emx1-Cre*^ mice compared to control mice (Fig. 2C, star and arrowhead), suggesting that the localization of neural cells might be affected upon *Trio* deletion. These results indicated that *Trio* deletion was involved in the disruption of both the number and the location of neural cells in developmental DG.

### *Trio* Deletion Results in Reduced Cell Proliferation and Ectopically Dividing Progenitors in DG

**Fig. 3:**
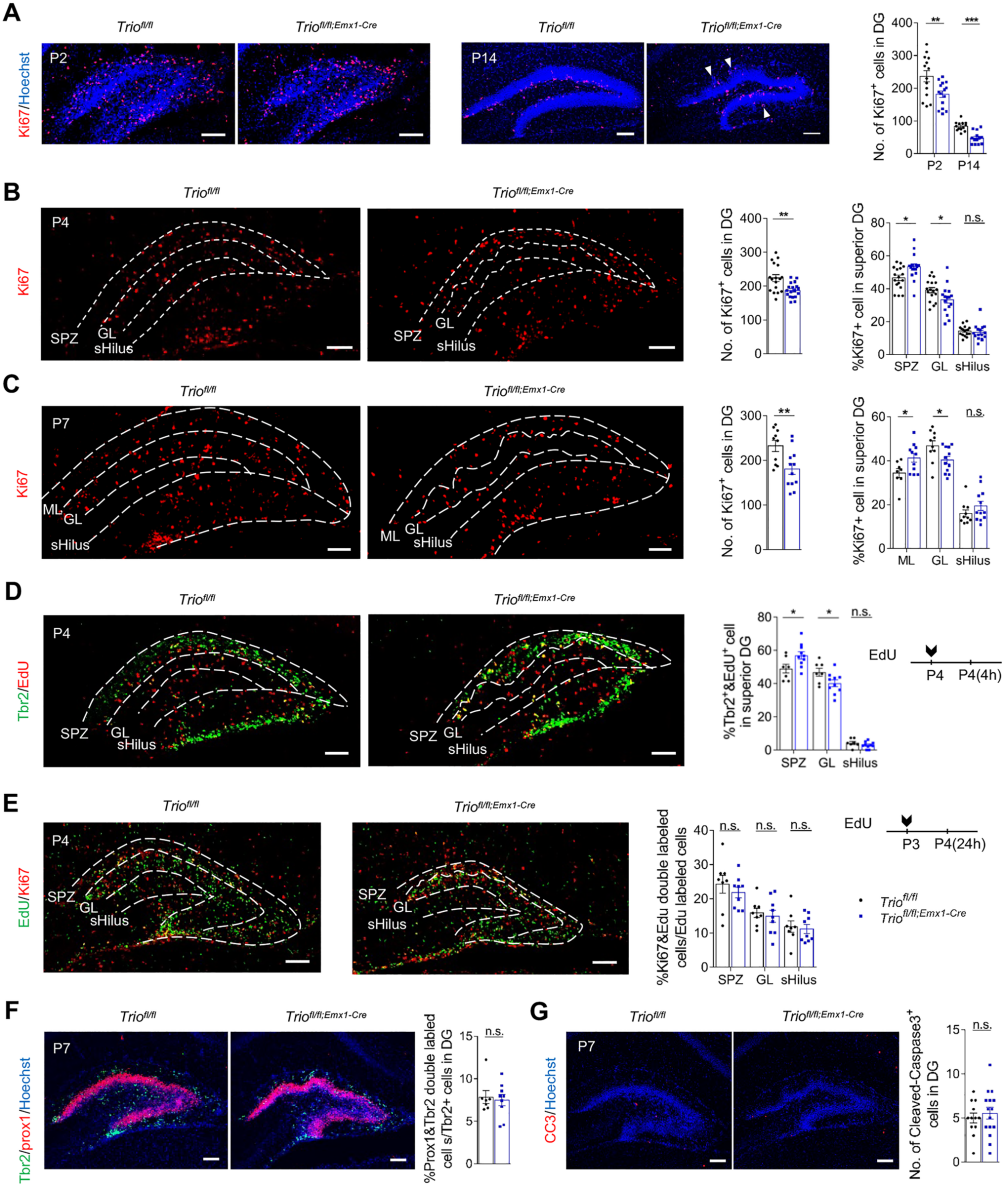
*Trio* deletion involves reduced and especially ectopic proliferation of IPCs in postnatal DG. **A** The absolute number of Ki67^+^ cells in DG at P2 (*n* = 4 WT; *n* = 4 cKO) and P14 (*n* = 4; 4). The arrowhead indicated the ectopic distributed Ki67^+^ cell in *Trio*^*fl/fl;Emx1-Cre*^ DG. Scale bars, 100 µm. **B** The absolute number of Ki67^+^ cells in DG, and their relative percentages in each part of the superior DG based on the total number of Ki67+ cells in the superior DG at P4 (*n* = 5; 5). Dashed lines illustrated the borderlines of the SPZ, GL, and superior parts of hilus. Scale bars, 100 µm. **C** The absolute number of Ki67^+^ cells in DG, and their relative percentages in each part of the superior DG based on the total number of Ki67+ cells in the superior DG at P7 (*n* = 3; 4). Dashed lines illustrated the borderlines of the ML, GL, and superior parts of hilus. Scale bars, 100 µm. **D** The relative percentages of proliferating IPCs in each part of the upper blade are based on the total number of Tbr2^+^EdU^+^ cells in the superior DG at P4 (*n* = 3; 4). Dashed lines illustrated the borderlines of the SPZ, GL, and superior parts of hilus. Mice were administrated EdU at P4 and sacrificed after 4 hours. Scale bars, 100 µm. **E** The relative number of cells existing cell-cycles in each part of the superior DG at P4 (*n* = 3; 3). Dashed lines illustrated the borderlines of the SPZ, GL, and superior parts of hilus. Mice were administrated EdU at P4 and sacrificed after 24 hours. Scale bars, 100 µm. **F** Relative percentages of the differentiating IPCs based on the total number of Tbr2^+^ cells in DG at P7 (*n* = 3; 3). Scale bars, 100 µm. **G** The absolute number of cleaved caspase-3^+^ cells in DG at P7 (*n* = 4; 4). Scale bars, 100 µm. Data were shown as means ± SEM. *P< 0.05, **P <0.01, ***P <0.001; n.s., no significance, two-tailed Student’s *t*-test.

The male morphogenesis of DG might be due to the abnormalities of several highly relative processes including cell proliferation, apoptosis, differentiation, and migration. The number of proliferating cells was found significantly reduced at P2, P4, P7, and P14 but not at P0 (Figs. 3A–C, S2A), confirming a postnatal hypoplasia. Moreover, the alteration was only found in the suprapyramidal but not the infrapyramidal blade (Fig. S2C–D). Notably, cells that proliferated in GL and ML, considered ectopic dividing cells, were increased in *Trio*^*fl/fl;Emx1-Cre*^ mice at P14, the end of proliferation peak of postnatal DG (Fig. 3A arrowhead, Fig. S2B), suggesting that the ectopic proliferation might be involved during early postnatal stages.

Since progenitors gathered in SPZ and then transferred into SGZ from P4 to P7, we then examined the distribution pattern of Ki67^+^ cells at P4 and P7. The results showed that the distribution proportion of Ki67^+^ cells was increased in the SPZ and ML at P4 and P7, respectively, but decreased in the GL of *Trio*^*fl/fl;Emx1-Cre*^ mice at both P4 and P7 (Fig. 3B, C). Considering that Tbr2^+^ IPCs were the largest population of proliferating cells in SPZ at P4, we then further confirmed whether the ectopic IPCs underwent abnormal mitosis in ectopic positions. Brain sections were stained with Tbr2 following a 4 h EdU injection at P4. We found that the proportion of proliferative IPCs increased in SPZ while decreased in GL in the superior DG of *Trio*^*fl/fl;Emx1-Cre*^ mice (Fig. 3D). Together with the results of pulse EdU labeling the number of proliferating cells was decreased in the superior mutant DG, but the proportion of EdU^+^ cells was increased in SPZ (Fig. S2E), it indicated that more IPCs would keep multiplying in SPZ rather than migrated to form SGZ in *Trio*^*fl/fl; Emx1-Cre*^ mice. Results found in the cell cycle exit assay performed in specific regions of DG at P4 showed that the ectopic IPCs could exit the cell cycle in mutant DG as those in control ones(Fig. 3E). Taken together, our data indicated that though the total number of proliferating cells was decreased in DG, especially in the superior part of DG, due to *Trio* ablation, IPCs accumulated and multiplied in the SPZ instead of settling into SGZ at early postnatal stages, which might contribute to the ectopic distribution of GCs.

Furthermore, we performed double immunostaining for Tbr2 and Prox1 at P7 to analyze the differentiation from IPCs to postmitotic neurons by calculating the proportion of Tbr2^+^Prox1^+^ double-labeled cells in total Tbr2^+^ cells. We found that the differentiation was not disturbed in *Trio*^*fl/fl;Emx1-Cre*^ mice (Fig. 3F). Also, the effect of apoptosis was precluded by the Cleaved Caspase-3 staining (Fig. 3G).

### *Trio* is Crucial for Neuron Migration in Postnatal DG Development

**Fig. 4:**
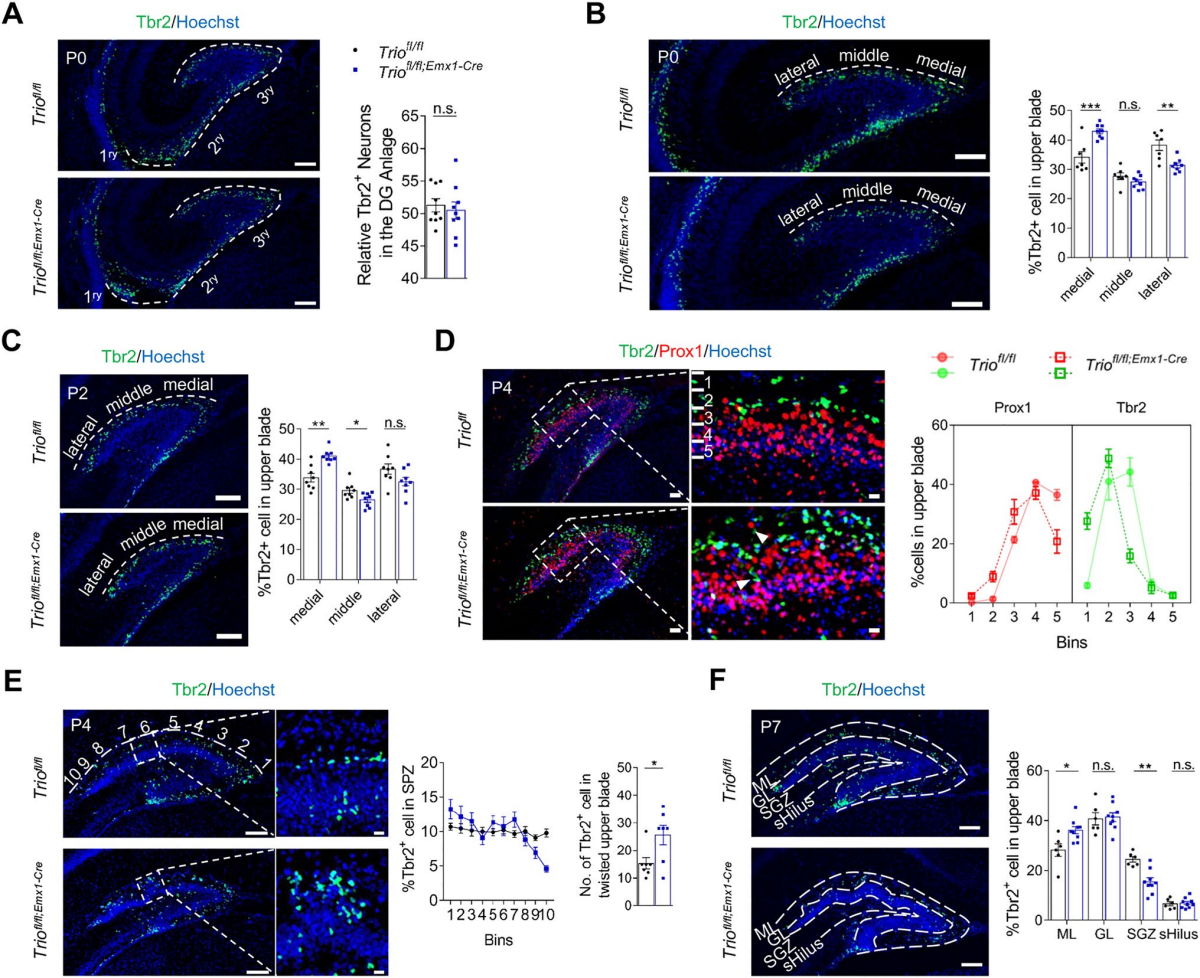
*Trio* is crucial for neuron migration in postnatal DG development. **A**–**C** Tangential migration of IPCs was evaluated by calculating the relative percentage of Tbr2+ cells in the DG anlage based on the total number of IPCs that migrated from neuroepithelium at P0 (**A**), and the proportions of IPCs in three equal parts of SPZ at P0 (**B**) and P2 (**C**) (*n* = 3 WT; *n* = 3 cKO). Scale bars, 100 µm. **D** Distribution pattern of IPCs and postmitotic neurons in SPZ was evaluated at P4 by dividing SPZ into five equal parts and calculating the proportions of Tbr2^+^ and Prox1^+^ cells in each part respectively (*n* = 3; 3). Arrowheads indicated the ectopic distributed cells in *Trio*^*fl/fl;Emx1-Cre*^ mice. Scale bars, 50 µm (left), 10 µm (right). **E** IPC migration in SPZ was evaluated by calculating the proportions of Tbr2^+^ cells in ten equal parts of SPZ, and the number of IPCs accumulated in the twisted upper blade at P4 in *Trio*^*fl/fl*^ mice and *Trio*^*fl/fl;Emx1-Cre*^ mice (*n* = 3; 3). Scale bars, 100 µm (left), 10 µm (right). **F** The SPZ-SGZ transition of IPCs was evaluated by Tbr2 labeling at P7 and their relative percentages in each part of the superior DG were calculated (*n* = 3; 3). Scale bars, 100 µm. Data were shown as means ± SEM.*P <0.05, **P <0.01, ***P <0.001; n.s., no significance, two-tailed Student’s *t*-test.

To assess the tangential migration of IPCs, we performed Tbr2 immunostaining and EdU labeling. We found that the percentage of Tbr2^+^ progenitors that arrived at the DG anlage was not dramatically changed in *Trio*^*fl/fl;Emx1-Cre*^ mice at P0 (Fig. 4A), which was further confirmed by the statistically equal percentage of Edu^+^ cells in the 3^ry^ in both genotypes(Fig. S3A). However, when the SPZ was divided into three equal parts medial, middle, and lateral part, we found that the percentage of Tbr2^+^ progenitors in the medial part was increased at both P0 and P2 (Fig. 4B, C). Furthermore, the total number of IPCs in SPZ was detected reduced at P2 and P4 (Fig. S3B), indicating that the formation of SPZ was damaged as a consequence of the reduction of tangential migration velocity of progenitors in *Trio*^*fl/fl;Emx1-Cre*^ mice.

To further characterize the migrating pattern in SPZ, double immunostaining for Tbr2 and Prox1 was performed. Unlike cells that were characterized with sharp boundaries in control mice, in *Trio*^*fl/fl;Emx1-Cre*^ mice, migratory streams of Tbr2^+^ and Prox1^+^ cells converged, so that more postmitotic cells located ectopically in SPZ, and more IPCs were crowded distributed beneath the pial surface, resulting Tbr2^+^ cells and Prox1^+^ cells were mixed up in local areas of the suprapyramidal blade (Fig. 4D, arrowhead). Thus, Tbr2^+^ IPCs accumulated focally in twisted parts in SPZ of *Trio*^*fl/fl;Emx1-Cre*^ mice (Fig. 4E). Taken together, these data suggested that the distribution patterns of neurons in SPZ were disrupted both tangentially and radially after the deletion of *Trio*.

To further detect the SPZ-SGZ transition of progenitors, the distribution proportion of Tbr2^+^ cells in each part of the suprapyramidal blade of DG was calculated at P7. The result showed that a greater percentage of IPCs were more likely to stay in ML, but not transit into SGZ in in *Trio*^*fl/fl;Emx1-Cre*^ mice, compared with control (Fig. 4F), while the IPCs in SGZ were distributed irregularly (Fig. S3C). To investigate further, we injected mice with EdU at P3 and harvested the brains at P7, that EdU^+^ cells migrating from SPZ gathered to form a symmetric cell band which would be the SGZ region. But in mutant SGZ, cells were distributed extremely asymmetrically, cells were even absent in some positions (Fig. S3D, arrowhead). These data suggested that the radial migration of IPCs was disrupted which resulted in a disturbance in the formation of SGZ in *Trio* ablated mice.

As neurons migrated along the scaffolds consisting of radial glia cells, we then examined the primary and secondary glia scaffolds by immunostaining the markers of radial glia cells during different developmental stages. BLBP and Vimentin staining showed normal density and extended long processes across the fimbria and radially to the hippocampal fissure at E16.5 and P0 (Fig. S4A, B) in *Trio*^*fl/fl;Emx1-Cre*^ mice, while the distribution and orientation of the radial glia scaffold were slightly changed in the DG anlage at P0 (Fig. S4B) in *Trio*^*fl/fl;Emx1-Cre*^ mice when comparing to controls. However, the secondary glia scaffold labeled with GFAP was missing above the twisted part of the GC band, and the orientation of the radial glia scaffold was also disrupted in *Trio*^*fl/fl;Emx1-Cre*^ mice at P7 and P14 (Fig. S4C, D).

In summary, our data showed that *Trio* deletion in DG led to the malformation of both SPZ and SGZ, indicating that Trio plays a critical role in neuron migration in postnatal DG development.

### *Trio* Regulates the Migration of Postmitotic Cells That Provides the Framework of Postnatal Reorganization of Dentate Neurons

**Fig. 5:**
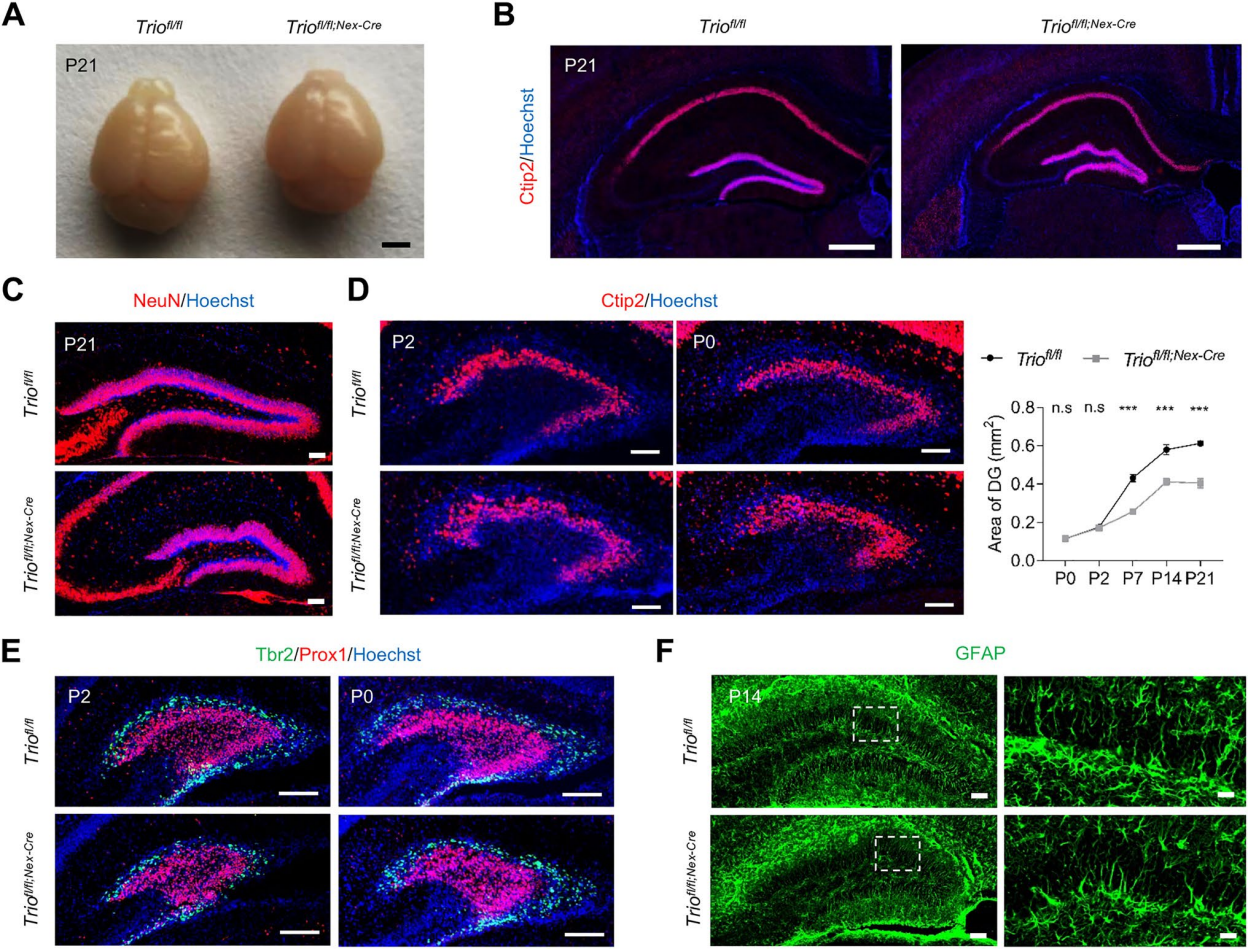
Similar but milder impairments were observed in postnatal DG of *Trio*^*fl/fl;Nex-Cre*^ mice. **A** Brain size of *Trio*^*fl/fl;Nex1-Cre*^ mice was smaller at P21. Scale bar, 2mm. **B** P21 coronal sections revealed abnormal morphology of DG in *Trio*^*fl/fl;Nex-Cre*^ mice. Scale bars, 100 µm. **C**-**D** Morphological changes in Trio-deleted DGs at postnatal developing stages (**C** and **D** left) and the area of the DG decreased in different levels in *Trio*^*fl/fl;Nex-Cre*^ DGs (**D** right) (*n* = 5 WT; *n* = 5 cKO). Data were shown as means ± SEM. ***P <0.001; n.s., no significance, two-tailed Student’s t-test. **E** Migration streams of Tbr2^+^ and Prox1^+^ cells were converged in *Trio*^*fl/fl;Nex-Cre*^ mice at P2, but not at P0. Scale bars, 100 µm. **F** The secondary radial glia scaffold was detected by GFAP staining at P14, and no impairments were found in *Trio*^*fl/fl;Nex-Cre*^ mice. Scale bars, 100 µm (left), 10 µm (right).

Since the localization of IPCs and postmitotic cells were both affected in *Trio*^*fl/fl;Emx1-Cre*^ mice, to further identify the type of neurons that play a dominant role in the postnatal morphogenesis of the suprapyramidal blade, we generated *Trio*^*fl/fl;Nex-Cre*^ mice, in which Trio was specifically knocked out in postmitotic excitatory neurons. In these mice, smaller brain size and reduced DG area were observed (Fig. 5A, D), with a remarkably twisted upper blade of DG (Fig. 5B). The GCs in *Trio*^*fl/fl;Nex-Cre*^ mice were also loosely and zigzagged packed (Fig. 5C). Meanwhile, converged migration streams of Tbr2^+^ and Prox1^+^ cells were detected in *Trio*^*fl/fl;Nex-Cre*^ mice at P2, suggesting a deficiency in neuron distribution and migration (Fig. 5E). However, when evaluating the glia scaffold of *Trio*^*fl/fl;Nex-Cre*^ DG by staining GFAP and Vimentin, no remarkable impairment was detected at any time point of postnatal development (Figs. 5F, S5A). In addition, the result of labeling proliferative cells by Ki67 at P14 showed that Ki67^+^ cells were reduced in the SGZ and ectopically distributed in the GCL and the ML of *Trio*^*fl/fl;Nex-Cre*^ mice (Fig. S5B), these results indicated a malformation of SGZ, which was similar to the deficiency in *Trio*^*fl/fl;Emx1-Cre*^ mice. According to the above results, similar impairments were observed in *Trio*^*fl/fl;Nex-Cre*^ mice, although the phenotype of twisted DG blades was milder than that in *Trio*^*fl/fl;Emx1-Cre*^ mice. Thus, we can infer that the damage in postmitotic cell migration was most associated with the malformation of postnatal DG.

### Spatial-Transcriptome Sequencing Revealed Different Functions of *Trio* in Various DG Excitatory Neurons

**Fig. 6:**
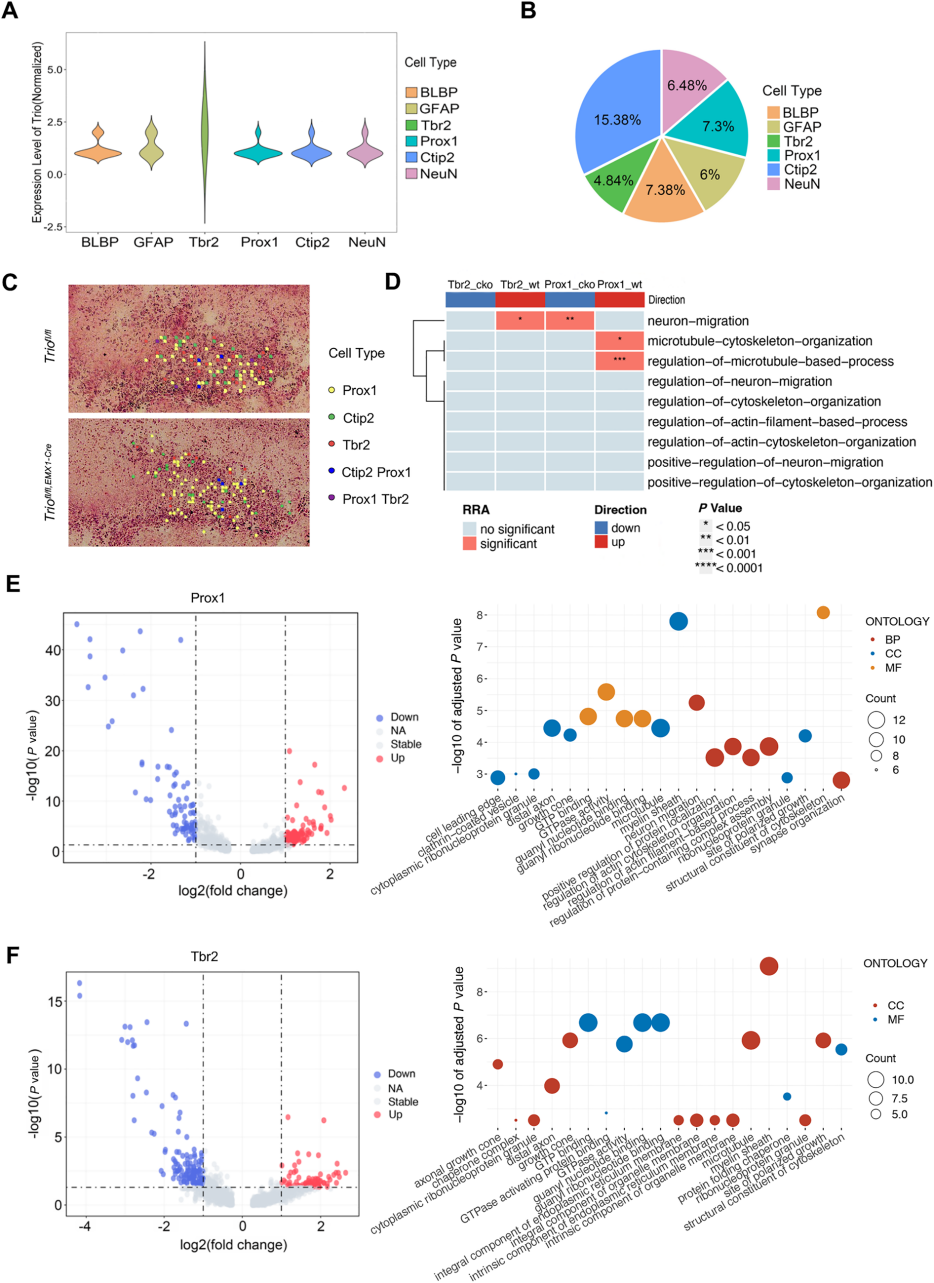
Spatial-transcriptome sequencing revealed different functions of *Trio* in various DG excitatory neurons. **A** Violin plot of the normalized expression level of Trio in different cell types of DG at P0 in spatial-transcriptome seq data with bin size of 30. **B** Relative percentages of cells expressing Trio based on the total detected numbers of each type of cell. **C** Spatial feature plots of prox1-, Tbr2- and CTIP2-expressing and prox1-Tbr2- and CTIP2-prox1-co-expressing cells in *Trio*^*fl/fl*^ and *Trio*^*fl/fl;Emx1-Cre*^ mice tissue sections. **D** GSEA analysis of Tbr2^+^ and prox1^+^ cells in neural migrating pathways. **E** Volcano plot of differentially expressed genes in spatial-transcriptome seq data form prox1^+^ cells in *Trio*^*fl/fl*^ and *Trio*^*fl/fl;Emx1-Cre*^ DGs (left). The bubble plot showed the top 20 terms of converged pathways that were down-regulated in *Trio*^*fl/fl;Emx1-Cre*^ DG by GO analysis (right). **F** Volcano plot of differentially expressed genes in spatial-transcriptome seq data form Tbr2^+^ cells in *Trio*^*fl/fl*^ and *Trio*^*fl/fl;Emx1-Cre*^ DGs (left). The bubble plot showed the top 20 terms of converged pathways that were down-regulated in *Trio*^*fl/fl;Emx1-Cre*^ DG by GO analysis (right).

To investigate the underlying molecular mechanisms in abnormal postnatal development of DG, we generated and analyzed the spatial-transcriptomic data using direct approaches to define and select cell populations. We isolated 6 subclasses of DG neural cells at P0, based on the well-known markers of NSCs, progenitor cells, and immature and mature GCs, which were BLBP^+^, GFAP^+^, Tbr2^+^, Prox1^+^, CTIP2^+,^ and NeuN^+^ cells. The expression level of Trio showed cell-specific diversity (Fig. 6A). At the cluster level, only 4.84% Tbr2^+^ cells expressed Trio, while 7.3% Prox1^+^ cells and 15.38% CTIP2^+^ cells expressed Trio (Fig. 6B). We performed additional confirmation by RNAscope co-staining of Trio with Tbr2 and CTIP2 respectively, and found that a higher Trio RNA expression level was detected in GCs rather than in progenitors (Fig. S6A).

Spatial-transcriptomic data was generated from a pair of *Trio*^*fl/fl*^ and *Trio*^*fl/fl;Emx1-Cre*^ mice brain at P0, which validated the ablation of Trio in DG by RNAscope (Fig. S6B). Alignment with single-cell bins data confirmed the spatial localization of excitatory neurons in the DG regions, and cells were isolated based on the expression of marker genes Tbr2, Prox1, and CTIP2 (Fig 6C). GSEA analysis was performed In Tbr2^+^ and Prox1^+^ cells in both *Trio*^*fl/fl;Emx1-Cre*^ mice and the control mice, showing that Prox1^+^ cells were highly involved in neuron migration and cytoskeleton organization and regulation, rather than Tbr2^+^ progenitor cells (Fig 6D). These results indicated that expression levels of *Trio* in these developing neuronal cells were variable, which led us to speculate on the variable roles that different neuronal cells play in postnatal DG development.

We then conducted differential gene expression analysis in progenitors, postmitotic cells, and radial glial cells respectively between *Trio*^*fl/fl;Emx1-Cre*^ mice and control. We found that *Trio* deletion in GFAP^+^ cells did not induce numerous genes down-regulating or up-regulating (Fig. S6C). However, Gene ontology (GO) analysis revealed that Trio was associated with neuron migration, regulation of reorganization of cytoskeleton components, and synapse organization among others in Prox1^+^ postmitotic neurons (Figs. 6E, S6D). In Tbr2^+^ progenitors, alteration of Trio was associated with the site of polarized growth, axonal growth, and GTP-protein activity (Figs. 6F, S6E). Thus, we can infer that *Trio* contributes to the postnatal reorganization of dentate GC blades by involving in diverse biological pathways in different cell populations, among which the migration of postmitotic neurons was directly regulated by *Trio*.

Furthermore, we accessed the expression level of reported ASD-related genes in the 6 subclasses of DG neural cells at P0 and found that the expression patterns of these genes were quite disparate in different developing neuronal cells of DG (Fig. S6F), suggesting a probable reason of the complicated etiology of ASD.

### *Trio*-Deleted Mice Exhibit Autism-like Behaviors

**Fig. 7:**
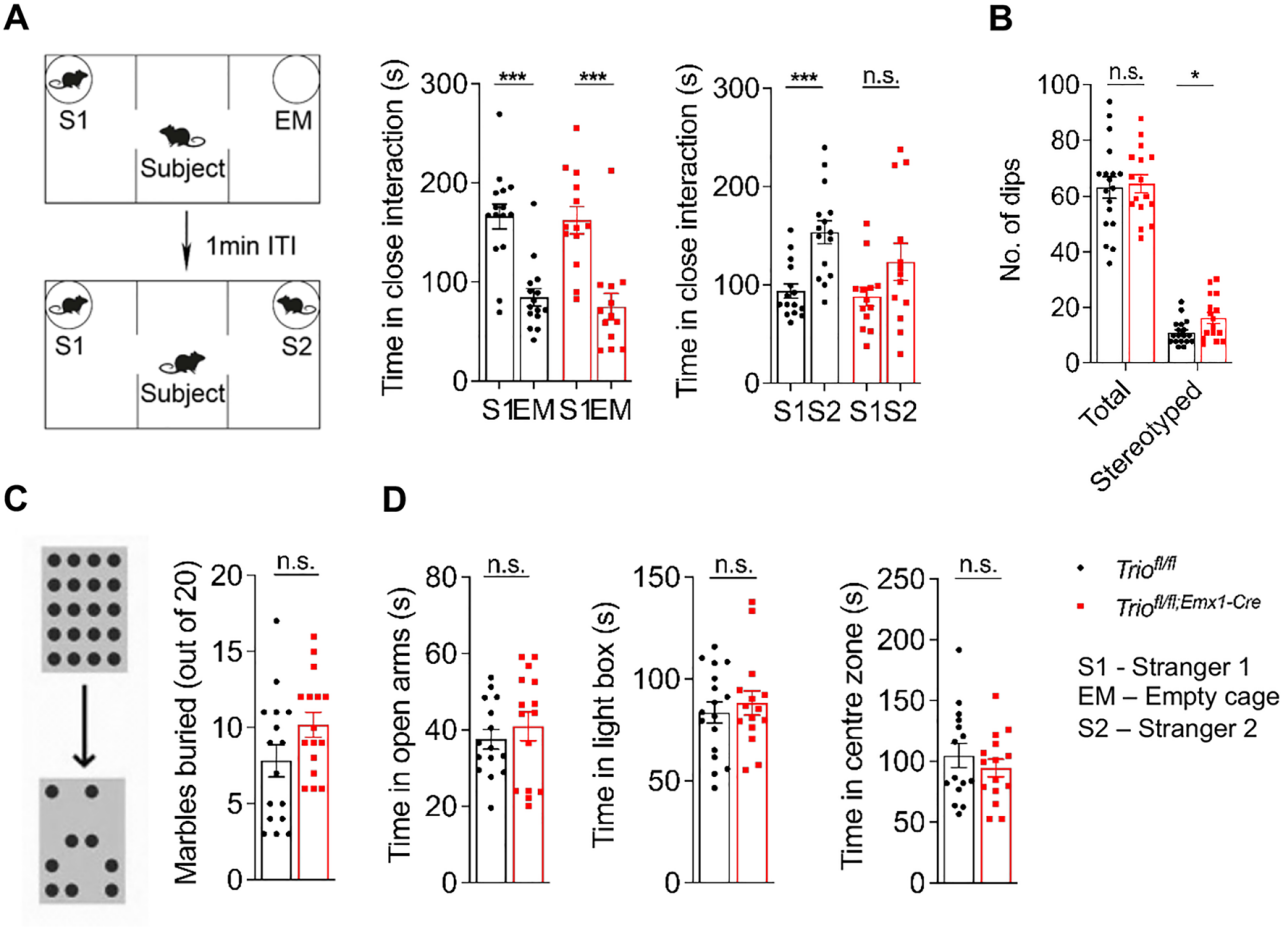
*Trio*-deletion-induced DG hypoplasia is related to autism-like behaviors. **A** Three-chamber social interaction (SI) test. In the sociability trial (S1 vs EM), mice of both genotypes spent more time in close interaction with strangers1 (S1) than in the empty chamber (EM). In the social novelty preference trial (S1 vs S2), *Trio*^*fl/fl;Emx1-Cre*^ mice did not display a preference for the novel social partner, stranger2 (S2) (*n* = 15 WT; *n* = 13 cKO). **B**-**C** Stereotype behaviors were detected by the hole-board test and marble burying test. *Trio*^*fl/fl;Emx1-Cre*^ mice performed more stereotyped digs (**B**, *n* = 18; 16) and buried more marbles (**C**, *n* = 16; 16). **D** Anxiety-like behaviors were determined by calculating the time spent in the open arm in the elevated plus-maze (*n* = 14; 14), the time spent in the light box in the light-dark box test (*n* = 17; 15), and the time spent in the center region during the first 10 min in the open field test (*n* = 15; 15). Data were shown as means ± SEM. *P <0.05, **P <0.01, ***P <0.001; n.s., no significance, two-tailed Student’s *t*-test.

To investigate the potential association of *Trio* deletion-induced DG malformation with autism-related abnormal behaviors, we conducted a battery of behavioral tests. In the three-chamber social interaction test, we found the deficiency of social novelty recognition in *Trio*^*fl/fl;Emx1-Cre*^ mice, while the social ability was not affected (Fig. 7A). Stereotyped behaviors were evaluated by hole-board test and marble burying test, showing that stereotyped number in *Trio*^*fl/fl;Emx1-Cre*^ mice were increased (Fig. 7B, C). However, the anxiety-like behaviors in *Trio*^*fl/fl;Emx1-Cre*^ mice tested by elevated plus-maze, light-dark box, and open field assay showed no difference compared to the control mice (Fig. 7D).

These results revealed that *Trio*^*fl/fl;Emx1-Cre*^ mice displayed social deficits and increased stereotyped behaviors, which were core abnormal behaviors related to ASDs.

## Discussion

In this study, we described dentate gyrus abnormalities in the knockout brains of *Trio*. The malformation of DG has been reported in some cases of autistic patients [10, 11] and mouse models [31–33], implying a close relationship between dysplasia of DG and autism behaviors. In a previous study [28], *Trio*^*fl/fl;Emx1-Cre*^ mice reported a smaller and disorganized DG and behavioral impairments in the Morris Water Maze and contextual fear conditioning test. However, in our research, we found that *Trio*^*fl/fl;Emx1-Cre*^ mice displayed autism-like behaviors including alteration of social behaviors and increased stereotyped behaviors, indicating that the malformation of the suprapyramidal blade was relevant to the development of ASD.

*Trio* is identified as a high-confidence ASD risk gene [24–27, 34]. Although Trio has been identified as an activator of small GTPases to regulate neuronal migration in different cell types of the cortex [19, 23, 35–37], its role in postnatal DG development was not well clarified [27]. We quantified the *Trio* expression level in these cells by spatial transcriptomic sequencing and RNAscope and noted that *Trio* was markedly increased during IPCs transferring into postmitotic cells. Moreover, *Trio* deficiency was associated with distinct alterations of signaling pathways in different cell populations, by regulating expression of diverse genes. In postmitotic neurons, Trio was related to biological processes of neuron migration and cytoskeleton organization, while in progenitors, Trio was more closely to the regulation of binding and GTPase activity. However, *Trio* deletion in GFAP^+^ cells did not induce numerous genes down-regulating or up-regulating, indicating that the role of *Trio* in GFAP^+^ cells was not critical during perinatal development. To our knowledge, this is the first study to provide evidence of *Trio* expression patterns and functions altering during the development of DG, providing a new insight into the etiology of *Trio*-loss-of-function-related ASD and other neurodevelopmental disorders. However, as neural stem cells and glial cells cannot be distinguished by marker genes at P0, we couldn’t figure out the expression pattern of *Trio* in NSCs, radial glial cells, and astrocytes exactly, which should be further researched in the future.

The developmental strategy of the postnatal DG is an orchestration of multiple neuronal cells including progenitors, immature GCs, and radial glial cells [38–40]. Abnormal hippocampal structure and disorderly arrangement of GCs in DG were reported in *Nestin-Cre* and *Emx1-Cre*-driven *Trio*-deletion mice during postnatal development stages [19, 28]. Consistent with previous studies, the deletion of *Trio* in *Trio*^*fl/fl;Emx1-Cre*^ and *Trio*^*fl/fl;Nex-Cre*^ mice also induced smaller brain size and twisted DG. Therefore, we can infer that impairment of the arrangement of postmitotic neurons, rather than neuronal stem cells and progenitors, is the main cause of the disorganized GC layer. However, the characteristics of disorganized granule cell layers were slightly different among these *Trio*-deletion strategies. In *Trio*^*fl/fl;Nestin-Cre*^ mice, the length of the suprapyramidal blade was remarkably shortened, and inflection was detected in the infrapyramidal blade [19]. While in *Trio*^*fl/fl;Emx1-Cre*^ mice, impairment was affected both supra-and infra-blades of DG, with more severe distortion in the upper blade. The *Trio*^*fl/fl;Nex-Cre*^ mice exhibited similar but milder defects in the arrangement of GC in DG. The malformation of DG anlage was detected in *Trio*^*fl/fl;Nestin-Cre*^ mice but not in *Trio*^*fl/fl;Emx1-Cre*^ and *Trio*^*fl/fl;Nex-Cre*^ DGs [41], but the loss of Prox1^+^ cells was observed at P0 in *Trio*^*fl/fl;Emx1-Cre*^ mice, thus we speculated that the prenatal developmental deficiency would exacerbate the malformation of postnatal DG. Moreover, spatial transcriptomic sequencing showed that the expression level and pathways involved in Trio vary in cell populations, making it reasonable that presentations of morphological and behavioral alterations would be diverse when Trio loses function in different developmental periods or cell types.

Furthermore, the radial scaffold formation was detected impaired in *Trio*^*fl/fl;Emx1-Cre*^ mice, but not in *Trio*^*fl/fl;Nex-Cre*^ mice, suggesting that the malformation of the radial glia scaffold exacerbated disorganization of GCs [42–44]. And intriguingly, abnormal migration of IPCs, caused by converged migrating streams of Tbr2^+^ cells and Prox1^+^ cells, in both *Trio*^*fl/fl;Emx1-Cre*^ mice and *Trio*^*fl/fl;Nex-Cre*^ mice, revealing that the ectopic distribution of progenitors was mainly induced by the abnormal migration of postmitotic GCs, instead of the cell-autonomous manner. Thus, our study provides new insights regarding the role of *Trio* in DG development and might help to understand its contribution to autism and other neurodevelopmental disorders.

## Supplementary Information

Below is the link to the electronic supplementary material.

Supplementary file1 (PDF 4361 kb)

## Data Availability

All data needed to evaluate the conclusions in the paper are present in the paper and/or the Supplementary Materials.
