# Supplementary material for: Dentate Gyrus Morphogenesis is Regulated by an Autism Risk Gene Trio Function in Granule Cells
Source: Neurosci Bull. 2024 Jun 22;41(1):1–15. doi: 10.1007/s12264-024-01241-y (PMC11748712; doi:10.1007/s12264-024-01241-y)
Supplement: Supplementary file 1 — Supplementary file1 (PDF 4361 kb) [file 12264_2024_1241_MOESM1_ESM.pdf]

Supplementary Materials

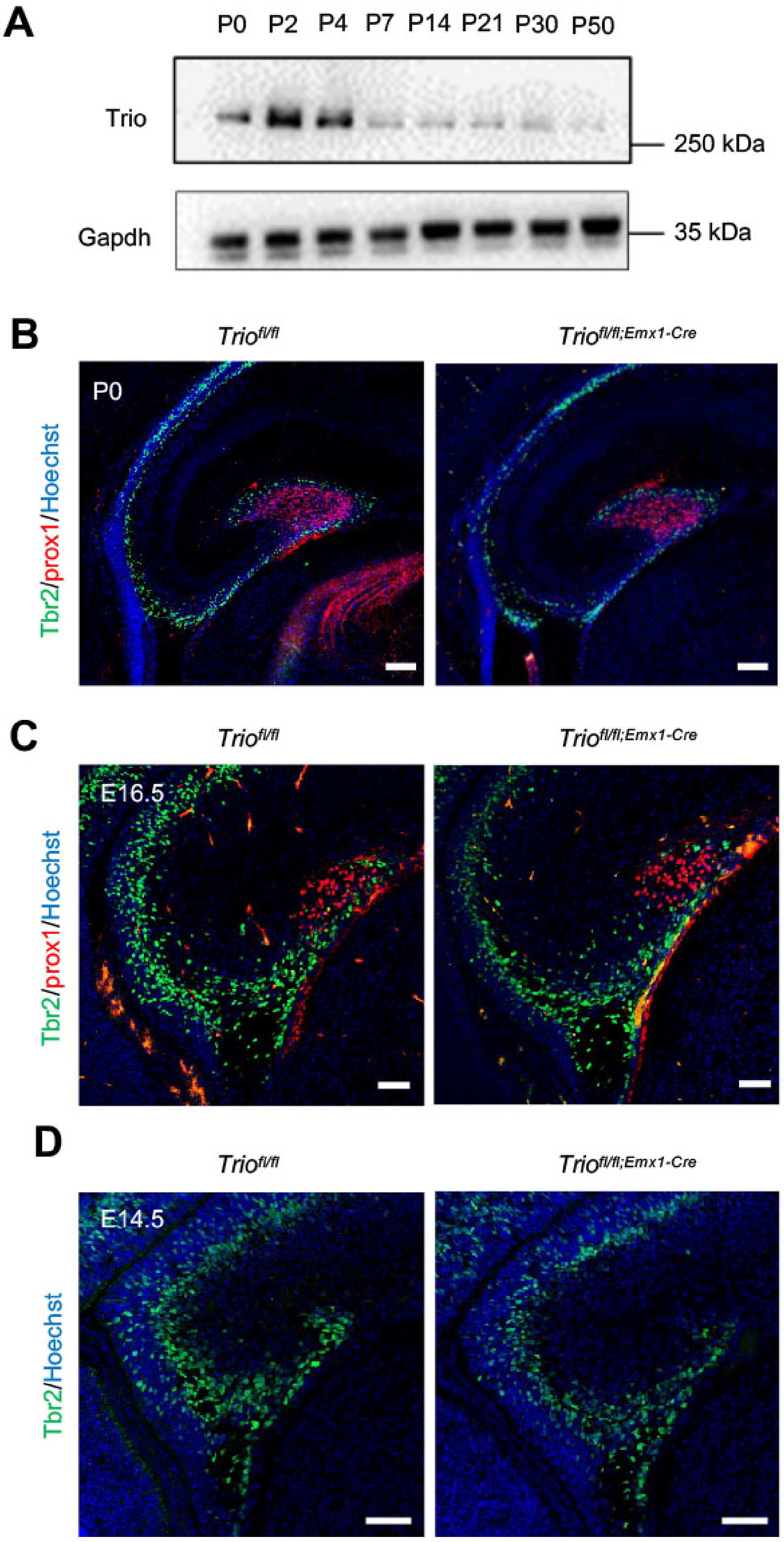

Fig. S1 The expression pattern of *Trio* in mouse postnatal hippocampus, and the morphology of DG anlage was normal at prenatal stages. **A** *Trio* protein expression level in HIP at different postnatal stages, that *Trio* was highly expressed during perinatal days, then decreased. **B–D** The spatial distribution of neurons positive for Tbr2 and prox1 in *Trio*$^{fl/fl}$ mice and *Trio*$^{fl/fl;Emx1\text{-}Cre}$ mice at P0 (**B**), E16.5 (**C**), E14.5 (**D**). Scale bars, 100 µm.

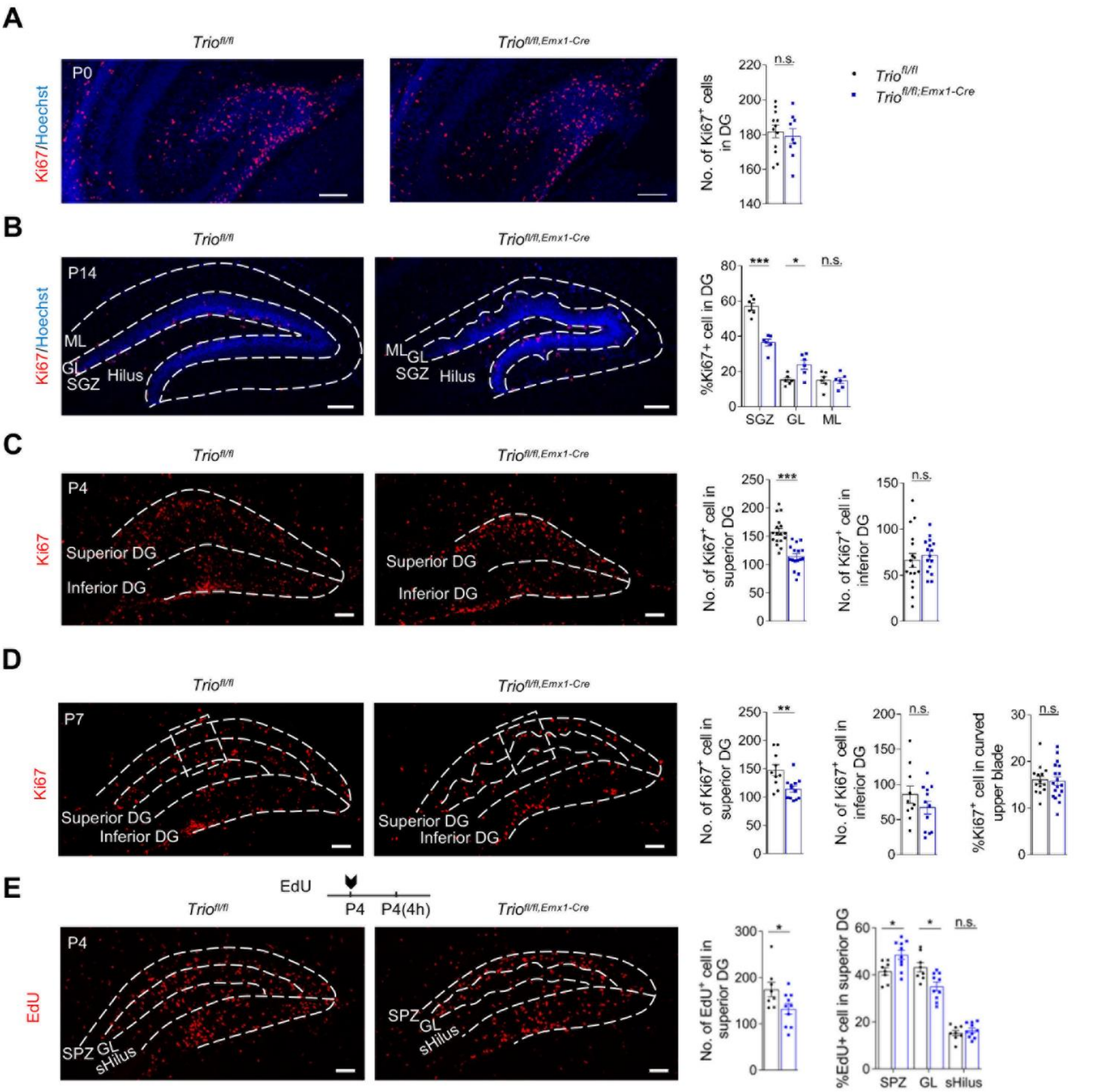

Fig. S2 Reduction of proliferating cells is mainly involved in the superior part but not the inferior part of postnatal DG in *Trio*$^{fl/fl;Emx1\text{-}Cre}$ mice. **A** The absolute number of Ki67^{+} cells in DG at P0 (n = 3 WT;

n = 3 cKO). Scale bars, 100 µm. **B** Relative percentages of Ki67^{+} cells in each part of DG based on the total number of Ki67+ cells in DG at P14 (n = 3; 3). Dashed lines illustrated the borderlines of the ML, GL, and hilus. Scale bars, 100 µm. **C** The absolute number of Ki67^{+} cells in superior and inferior DG at P4 (n = 5; 5). Dashed lines illustrated the borderlines of the superior and inferior parts of DG. Scale bars, 100 µm. **D** The absolute number of Ki67^{+} cells in superior and inferior DG at P7, and relative percentages in curved parts of the upper blade based on the total number of Ki67+ cells in the superior DG (n = 3; 3). Dashed lines illustrated the borderlines of the ML, GL, and shilus. Frames showed the curved part of the suprapyramidal blade. Scale bars, 100 µm. **E** The absolute number of EdU^{+} cells in superior DG at P4, and their relative percentages in each part of the upper blade based on the total number of EdU^{+} cells in the superior DG (n = 3; 4). Mice were administrated EdU at P4 and sacrificed after 4 hours. Dashed lines illustrated the borderlines of the ML, GL, and hilus. Scale bars, 100 µm. Data were shown as means ± SEM.*P <0.05, **P <0.01, ***P <0.001; n.s., no significance, two-tailed Student's t-test.

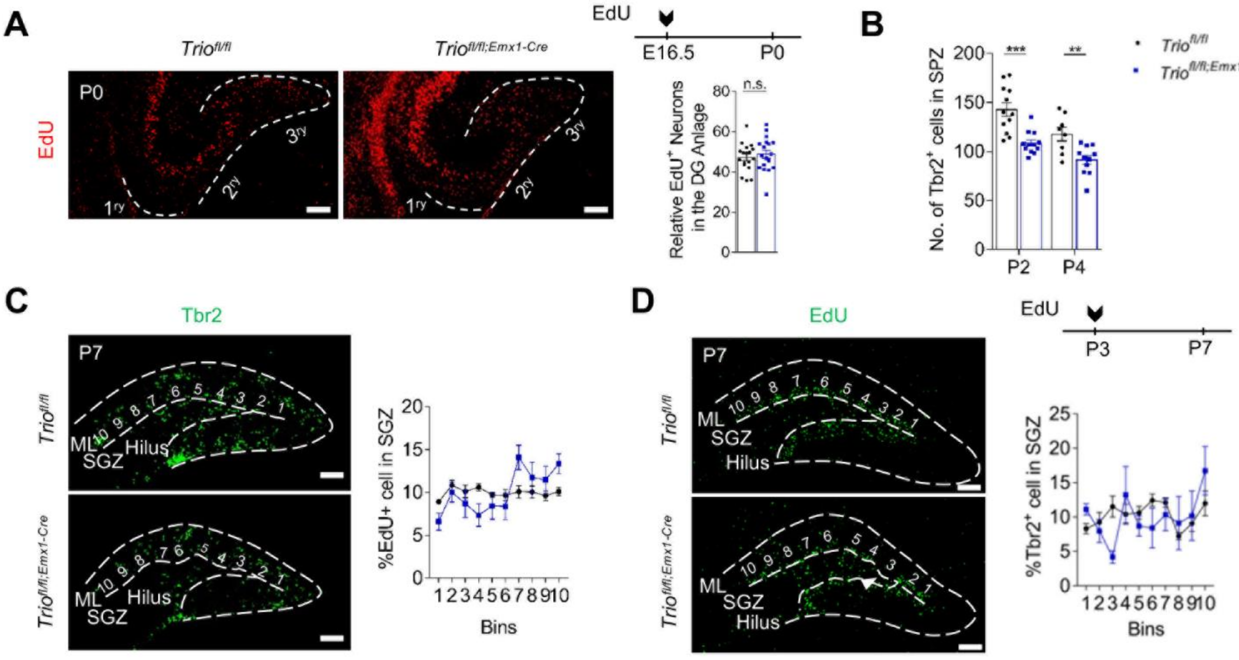

Fig. S3 *Trio* deletion impairs the formation of both SPZ and SGZ in postnatal DG but barely influences the tangential migration in the embryonic stage. **A** Normal tangential migration was observed in *Trio*$^{fl/fl;Emx1\text{-}Cre}$ DG in the embryonic stage, by injecting EdU at E16.5 and cells were harvested at P0 to evaluate the relative percentage of EdU^{+} cell in the DG anlage (n = 4 WT; n = 4 cKO). Scale bars, 100

μm. **B** The absolute number of Tbr2$^+$ cells in SPZ at P2 (n = 4; 4) and P4 (n = 3; 3). **C** The proportions of Tbr2$^+$ cells in ten equal parts of SGZ at P7 (n = 3; 3). Dashed lines illustrated the borderlines of the ML and SGZ. Scale bars, 100 μm. **D** The proportions of EdU$^+$ cells in ten equal parts of SGZ. Mice were administrated EdU at P3 and sacrificed at P7 (n = 3; 3). Dashed lines illustrated the borderlines of the ML and SGZ. Scale bars, 100 μm. Data were shown as means ± SEM. $^{**}P$ <0.01, $^{***}P$ <0.001; n.s., no significance, two-tailed Student's t-test.

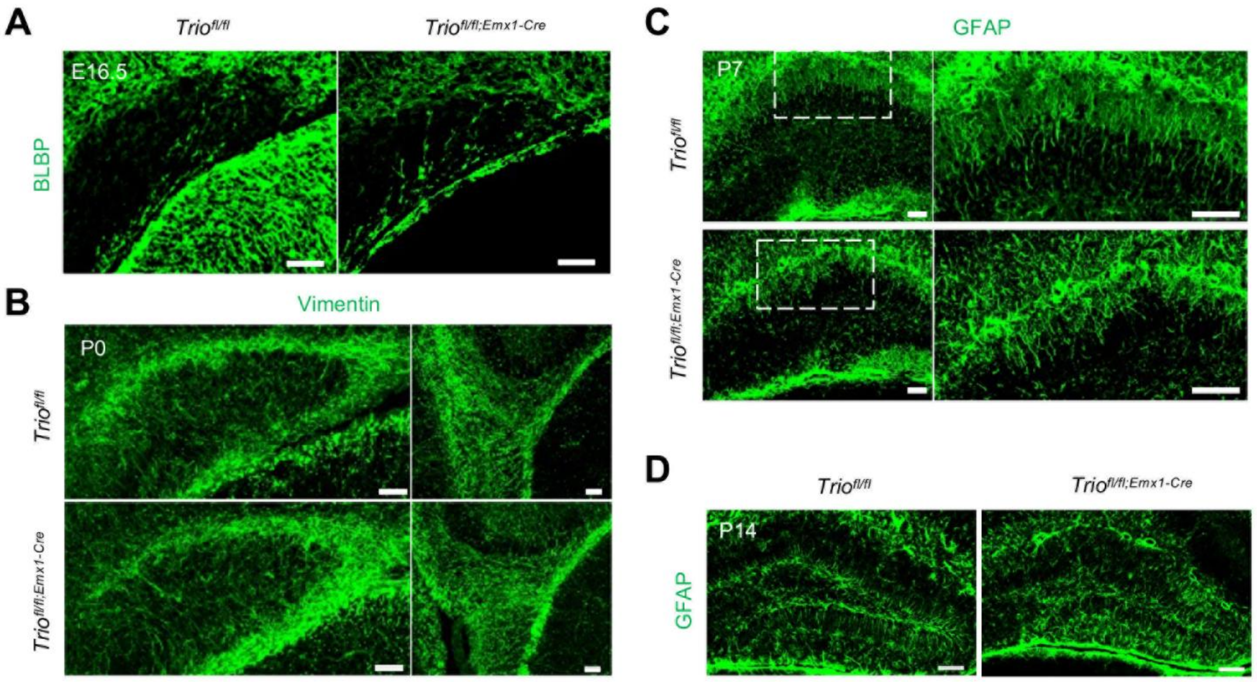

Fig. S4 The formation of the primary radial glia scaffold was not impaired, but defects of the secondary radial glia scaffold were detected in *Trio*-deletion mice. **A–B** The primary radial glia scaffold was detected by BLBP staining at E16.5 (**A**) and Vemintin staining at P0 (**B**). Scale bars, 20 μm (**A**), 50 μm (**B**). **C–D** The secondary radial glia scaffold was detected by BLBP staining at P7 (**C**) and P14 (**D**). Scale bars, 50 μm (**C**), 100 μm(**D**).

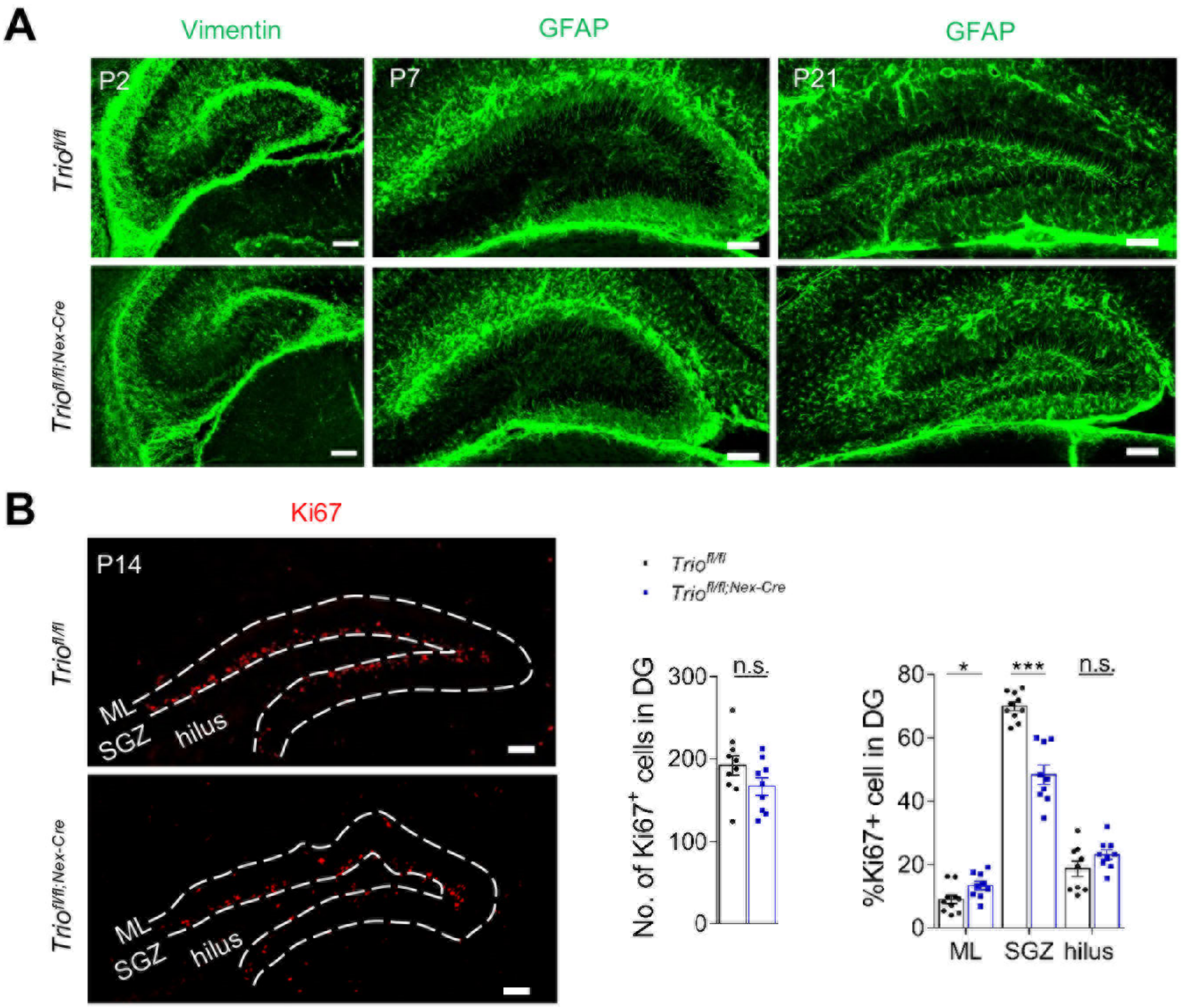

Fig. S5 Ectopic proliferation was detected in *Trio*$^{fl/fl;Nex\text{-}Cre}$ mice without deficits in radial glia scaffolds. **A** Radial glia scaffold was detected by Vimentin and GFAP staining at different postnatal developmental stages in *Trio*$^{fl/fl;Nex\text{-}Cre}$ and *Trio*$^{fl/fl;Nex\text{-}Cre}$ mice were normal. Scale bars, 100 µm. **B** The total number of Ki67$^+$ cells was not changed in *Trio*$^{fl/fl;Nex\text{-}Cre}$ mice at P21, but the proportion of Ki67$^+$ cells in SGZ was decreased and ectopic Ki67$^+$ cells were detected in ML (n = 3 WT; n = 3 cKO). Dashed lines illustrated the borderlines of the ML and SGZ. Scale bars, 100 µm. Data were shown as means ± SEM. **P* <0.05, ****P* <0.001; n.s., no significance, two-tailed Student's *t*-test.

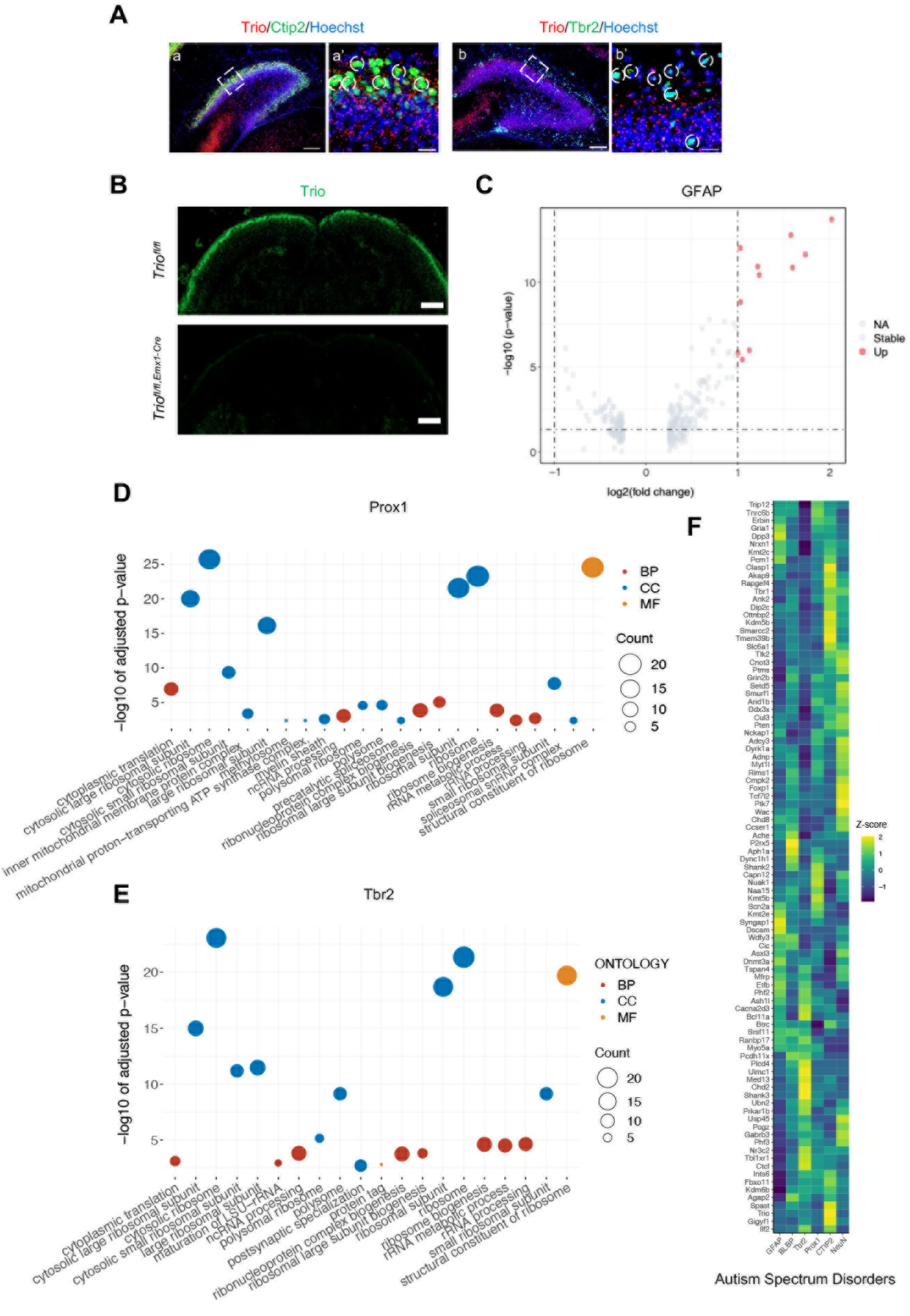
A
Trio/Ctip2/Hoechst
Trio/Tbr2/Hoechst
a
a'
b
b'
B
Trio
Trio$^{fl/fl}$
Trio$^{fl/fl,Emx1-Cre}$
C
GFAP
−log10 (p−value)
log2(fold change)
NA
Stable
Up
D
Prox1
−log10 of adjusted p−value
BP
CC
MF
Count
cytoplasmic translation
cytosolic large ribosomal subunit
cytosolic ribosome
cytosolic small ribosomal subunit
inner mitochondrial membrane protein complex
large ribosomal subunit
mitochondrial proton−transporting ATP synthase complex
methylosome
myelin sheath
ncRNA processing
polysomal ribosome
polysome
precatalytic spliceosome
ribonucleoprotein complex biogenesis
ribosomal large subunit biogenesis
ribosomal subunit
ribosome
ribosome biogenesis
rRNA metabolic process
rRNA processing
small ribosomal subunit
spliceosomal snRNP complex
structural constituent of ribosome
E
Tbr2
−log10 of adjusted p−value
ONTOLOGY
BP
CC
MF
Count
cytoplasmic translation
cytosolic large ribosomal subunit
cytosolic ribosome
cytosolic small ribosomal subunit
large ribosomal subunit
maturation of LSU−rRNA
ncRNA processing
polysomal ribosome
polysome
postsynaptic specialization
protein tag
ribonucleoprotein complex biogenesis
ribosomal large subunit biogenesis
ribosomal subunit
ribosome
ribosome biogenesis
rRNA metabolic process
rRNA processing
small ribosomal subunit
structural constituent of ribosome
F
Z-score
GFAP
BLBP
Tbr2
Prox1
CTIP2
NeuN
Autism Spectrum Disorders

Fig. S6 Spatial-transcriptome sequencing indicates the complicated etiology of *Trio* deletion-induced DG hypoplasia. **A** RNAscope co-staining of Trio with Tbr2 and Ctip2 respectively at P4 in $Trio^{fl/fl}$ mice. Scale bars, 100 µm (a, b), 10 µm (a', b'). **B** RNAscope of Trio in $Trio^{fl/fl}$ and $Trio^{fl/fl;Emx1\text{-}Cre}$ mice at P0. Scale bars, 500 µm. **C** Volcano plot of differentially expressed genes in spatial-transcriptome seq data form $GFAP^{+}$ cells in $Trio^{fl/fl}$ and $Trio^{fl/fl;Emx1\text{-}Cre}$ DGs. **D–E** Bubble plots showed the top 20 terms of converged pathways by GO analysis that were up-regulated in $Prox1^{+}$ (**D**) and $Tbr2^{+}$ (**E**) cells of $Trio^{fl/fl;Emx1\text{-}Cre}$ DG, respectively. **F** Heatmap showed the expression level of reported ASD-related genes in the 6 subclasses of DG neural cells at P0.
